# DENR–MCTS1 heterodimerization and tRNA recruitment are required for translation reinitiation

**DOI:** 10.1371/journal.pbio.2005160

**Published:** 2018-06-11

**Authors:** Yasar Luqman Ahmed, Sibylle Schleich, Jonathan Bohlen, Nicolas Mandel, Bernd Simon, Irmgard Sinning, Aurelio A. Teleman

**Affiliations:** 1 Heidelberg University Biochemistry Center (BZH), Heidelberg, Germany; 2 German Cancer Research Center (DKFZ), Heidelberg, Germany; 3 Heidelberg University, Heidelberg, Germany; 4 European Molecular Biology Laboratory (EMBL), Structural and Computational Biology Unit, Heidelberg, Germany; 5 National Center for Tumor Diseases (NCT), Heidelberg, Germany; Yale University, United States of America

## Abstract

The succession of molecular events leading to eukaryotic translation reinitiation—whereby ribosomes terminate translation of a short open reading frame (ORF), resume scanning, and then translate a second ORF on the same mRNA—is not well understood. Density-regulated reinitiation and release factor (DENR) and multiple copies in T-cell lymphoma-1 (MCTS1) are implicated in promoting translation reinitiation both in vitro in translation extracts and in vivo. We present here the crystal structure of MCTS1 bound to a fragment of DENR. Based on this structure, we identify and experimentally validate that DENR residues Glu42, Tyr43, and Tyr46 are important for MCTS1 binding and that MCTS1 residue Phe104 is important for tRNA binding. Mutation of these residues reveals that DENR-MCTS1 dimerization and tRNA binding are both necessary for DENR and MCTS1 to promote translation reinitiation in human cells. These findings thereby link individual residues of DENR and MCTS1 to specific molecular functions of the complex. Since DENR–MCTS1 can bind tRNA in the absence of the ribosome, this suggests the DENR–MCTS1 complex could recruit tRNA to the ribosome during reinitiation analogously to the eukaryotic initiation factor 2 (eIF2) complex in cap-dependent translation.

## Author summary

Usually, eukaryotic ribosomes translate only a single open reading frame (ORF) on an mRNA and then dissociate from the mRNA. In some cases, when there is a short upstream open reading frame (uORF) that precedes the main ORF, ribosomes can translate the uORF, terminate translation, and then undergo a poorly understood process called “translation reinitiation” whereby they resume scanning for another AUG initiation codon and then translate the main ORF. The molecular functions required for translation reinitiation are not known. We previously showed that two noncanonical initiation factors, density-regulated reinitiation and release factor (DENR) and multiple copies in T-cell lymphoma-1 (MCTS1), are involved in this process. We show here, based on a structure of MCTS1 bound to a fragment of DENR, that in order to successfully promote translation reinitiation, DENR and MCTS1 need to dimerize, and they need to bind tRNA. We thereby identify two molecular functions needed for translation reinitiation.

## Introduction

Eukaryotic translation reinitiation is a process that is only recently becoming understood at the mechanistic and functional levels. Unlike prokaryotic ribosomes, eukaryotic ribosomes were thought to engage mainly in a single round of initiation, extension, and termination on an individual mRNA. After recruitment of the ribosomal 40S subunit to the mRNA, often via the 5′ cap, the 40S scans to locate the first appropriate AUG start codon for joining of the 60S subunit and commencement of translation. After translating this open reading frame (ORF) and terminating, the 60S subunit dissociates from the mRNA, leaving the 40S subunit bound to the mRNA. In most cases, the 40S subunit then also dissociates from the mRNA, leaving additional downstream ORFs on the mRNA untranslated. However, when the translated ORF is short, as is the case for many upstream open reading frames (uORFs), the 40S can remain bound to the mRNA, resume scanning, and reinitiate translation at a downstream ORF [1]. Indeed, recent ribosome profiling studies have found pervasive translation of uORFs [2–4], indicating that in many of these cases, translation reinitiation is important for permitting translation of the main ORF on the mRNA.

Both standard cap-dependent translation initiation and translation reinitiation consist of a succession of events that are carefully orchestrated to result in initiation of translation. In the case of cap-dependent translation, this has been carefully studied and is known to involve the recruitment of tRNA via the ternary complex to the small ribosomal subunit yielding the 43S preinitiation complex (PIC) and the subsequent recruitment of this complex to the mRNA cap via the eukaryotic initiation factor 4F (eIF4F) complex. In the case of translation reinitiation, however, the succession of events is not yet clear. In particular, it is not known which factors are responsible for recruiting a new initiator tRNA to the ribosome and how this occurs.

**Fig 1:**
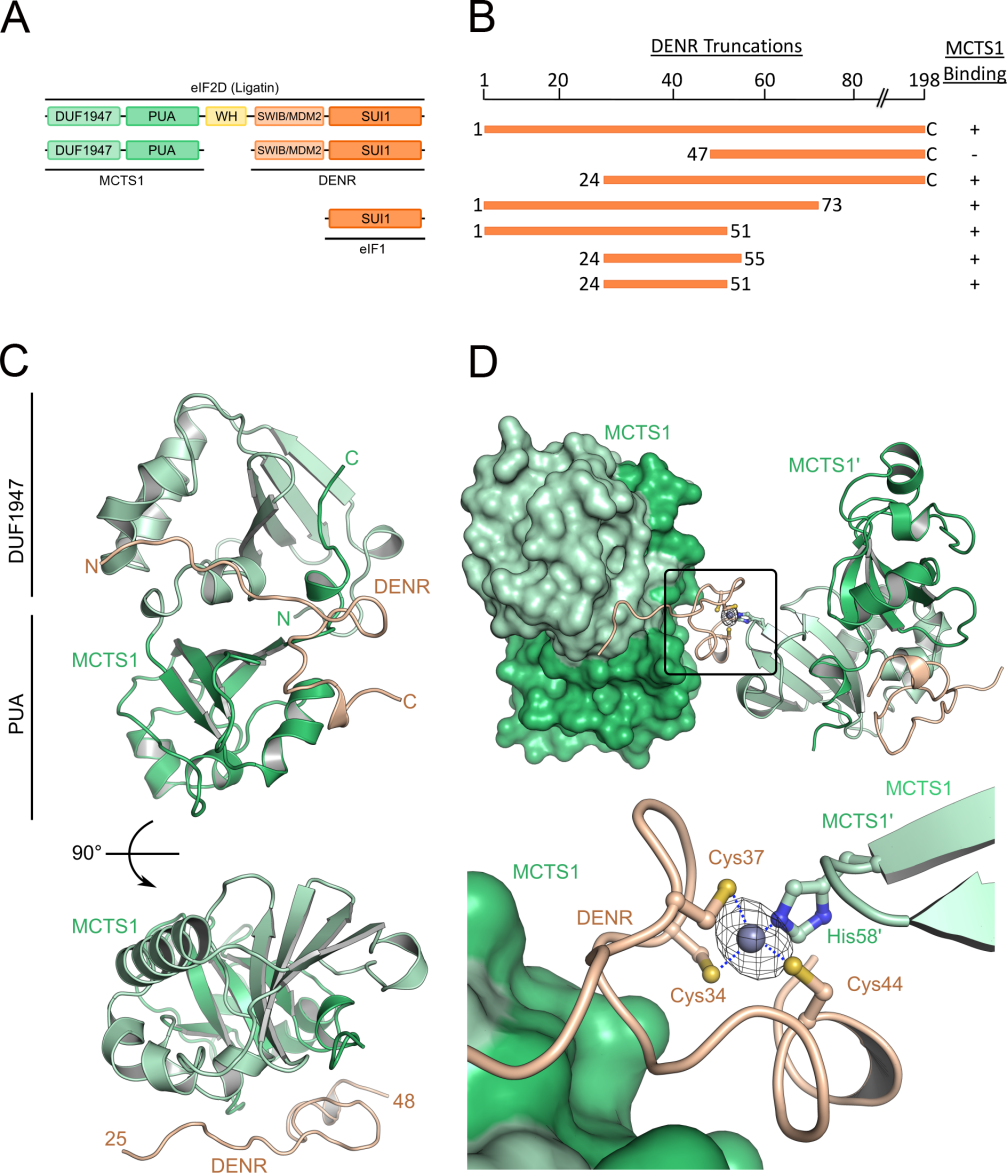
High-resolution structure of MCTS1 binding an N-terminal fragment of DENR. (A) Domain architecture of DENR, MCTS1, and the related eIF2D (ligatin) protein. (B) A DENR truncation series identifies aas 24–51 of DENR as the minimum peptide capable of binding MCTS1 in *Escherichia coli*. Summary of data presented in S1 Fig. (C) Crystal structure of the minimal DENR–MCTS1 complex. MCTS1 contains an N-terminal DUF1947 (light green) and a C-terminal PUA domain (green). The DENR peptide (light orange) binds along the interface between the N- and C-terminal MCTS1 domains. Anomalous density (14σ), calculated with ANODE, is shown as a mesh. (D) DENR contains a zinc finger at the N-terminus, comprised of Cys34, 37, and 44. Although our DENR construct lacks a fourth residue (Cys53) to complete the Zn^2+^ coordination, the zinc finger is properly folded, as it is completed by His58 of a crystallographically related MCTS1 molecule. aa, amino acid; DENR, density-regulated reinitiation and release factor; DUF1947, domain of unknown function 1947; eIF1, eukaryotic initiation factor 1; eIF2D, eukaryotic initiation factor 2D; MCTS1, multiple copies in T-cell lymphoma-1; MDM2, mouse double minute 2; PUA, pseudouridine synthase and archaeosine transglycosylase; SUI1, suppressors of initiation codon mutations 1; SWIB *SWItch*/Sucrose Nonfermentable complex B; WH, winged helix

Several factors have been implicated in promoting the reinitiation process. One such factor is eukaryotic initiation factor 3 (eIF3), which remains associated with ribosomes after termination on short uORFs [5]. Recent work has also implicated the proteins density-regulated reinitiation and release factor (DENR), multiple copies in T-cell lymphoma-1 (MCTS1), and eukaryotic initiation factor 2D (eIF2D) in reinitiation [6–10]. MCTS1 was first identified as a gene that is amplified at the genomic level in T-cell leukemias [11]. Subsequent studies found that MCTS1 protein levels are also elevated in T-cell lymphoid cell lines, in non-Hodgkin lymphoma cell lines, and in 85% of primary diffuse large B cell lymphomas [12]. Functional studies, mainly by the Gartenhaus lab, showed that MCTS1 has oncogenic properties. MCTS1 promotes anchorage-independent growth of NIH3T3 and MCF-10A cells [11,13], and its overexpression accelerates the cell cycle, shortening G1 and increasing cyclin D1 and cyclin-dependent kinase 4 (Cdk4) activities [14]. The DENR protein binds MCTS1 [15] and is overexpressed in breast cancer cells [16]. eIF2D is a larger protein that has sequence homology to MCTS1 in its N-terminal half and to DENR in its C-terminal half (Fig 1A), and functional studies suggest it combines the activities of MCTS1 and DENR [6,7]. To reinitiate translation, ribosomes need to reacquire an initiator tRNA. eIF2D or the combination of DENR–MCTS1 were found to promote eukaryotic initiation factor 2 (eIF2)-independent and guanosine triphosphate (GTP)-independent recruitment of Met-tRNA_i_^Met^ to 40S complexes if the initiation codon is positioned in the P-site of the 40S, as is the case for hepatitis C virus-like internal ribosome entry sites (IRESs) [6,7]. Indeed, eIF2D was shown to promote translation reinitiation when added to in vitro–reconstituted systems [8,9]. We recently showed that DENR and MCTS1 (also known as MCT-1) promote translation reinitiation on cellular mRNAs in *Drosophila* and in human cells [10,17].

The structure of MCTS1 contains a pseudouridine synthase and archaeosine transglycosylase (PUA) domain fold typically present in proteins that bind RNA [26]. DENR contains an SUI1 domain, similar to eukaryotic initiation factor 1 (eIF1) [7]. To gain further insights into the structure and function of the MCTS1–DENR complex, we set out to obtain a high-resolution structure of MCTS1 bound to DENR. Here, we present a 2.14 Å–resolution crystal structure of MCTS1 bound to an N-terminal fragment of DENR, which complements two recent low-resolution structures of eIF2D or DENR–MCTS1 bound to the 40S ribosomal subunit [19,20]. Our structure identifies specific residues on DENR and on MCTS1 important for heterodimerization of the complex and for tRNA binding, respectively. We find that mutation of these residues leads to strong functional impairments, reducing the ability of the DENR–MCTS1 complex to reinitiate translation in human cells. This shows that DENR–MCTS1 heterodimerization and tRNA binding are both required for this complex to promote translation reinitiation in vivo. Furthermore, we find that the DENR–MCTS1 complex is able to bind tRNA in the absence of the ribosome. This suggests the DENR–MCTS1 complex can first bind tRNA, forming a trimeric complex, and then recruit it to the ribosome during reinitiation, analogously to the eIF2 complex in cap-dependent translation.

## Results

### Identification of the DENR minimal MCTS1-binding domain

MCTS1 and DENR are homologous to the N- and C-terminal domains of eIF2D (also known as ligatin), respectively (Fig 1A), and they bind each other in vivo [15]. To study the binding interface between MCTS1 and DENR, we first aimed to identify the part of DENR responsible for MCTS1 binding. We coexpressed a polyhistidine (HIS)-tagged MCTS1 with untagged full-length DENR in *E*. *coli* and found that affinity purification of HIS-MCTS1 also pulls down DENR from bacterial cell lysates (lanes 1–7, S1A Fig). Using this binding assay and a series of DENR truncations, we identified DENR amino acids (aas) 24–51 as the minimal region for MCTS1 binding (Fig 1B and S1A–S1E Fig).

### Crystal structure of the minimal DENR–MCTS1 complex

A previous study reported the crystal structure of MCTS1 containing three mutations (E137A, K139A, and Q140A, henceforth called MCTS1x) that were designed to allow crystallization [18]. To determine the structure of DENR–MCTS1, we tried crystallizing the full-length heterodimeric complex, either with or without these three mutations, but we did not obtain crystals. Instead, we assembled and crystallized the minimal complex consisting of full-length wild-type (WT) MCTS1 and residues 24–51 of DENR and determined its crystal structure to 2.14-Å resolution (Fig 1C and 1D). Data collection and refinement statistics are summarized in Table 1. The asymmetric unit contains one DENR–MCTS1 complex, which has been defined to contain one biologically relevant assembly, instead of a complex that satisfies the metal ion coordination (see next paragraph). Although we initially determined the structure by molecular replacement, strong positive density near MCTS1-His58 and the three Cys residues of DENR suggested the presence of a metal ion, most likely zinc. We therefore collected a dataset at the Zn-edge and could determine the structure de novo by means of Zn–single-wavelength anomalous dispersion (SAD). The identity of zinc was confirmed by X-ray fluorescence (XRF) analysis (S1F Fig) and via a colorimetric assay using 4-(2-pyridylazo)resorcinol (PAR) [21,22] (S1G Fig). The overall structure of MCTS1 is essentially the same as the previously reported crystallization variant MCTS1x, as indicated by the low root-mean-square deviation (RMSD) of 0.71 Å for 181 residues (S1H Fig). The DENR N-terminus (residues 25 to 33) is largely extended, lacking secondary structure, while the C-terminal part contains a partial zinc finger (residues 34 to 44) and a short α-helix (residues 44 to 46), which, according to secondary structure prediction, would extend to residue 60. We superposed our structure onto MCTS1 in the recently published low-resolution structure of the 40S–DENR–MCTS1 complex [20]. The DENR-peptide from our heterodimer fits well into the unassigned electron density near the MCTS1 PUA domain (S1I Fig). The density for DENR residues 29–31 is not continuous, suggesting some flexibility in binding. In contrast, the density for the zinc finger is very well defined, and additional density is visible at the C-terminus of our construct, in agreement with the predicted α-helix.

**Table 1:** Data collection and refinement statistics.

	DENR–MCTS1 Zn-SAD
**Data collection**	
Beamline	ESRF ID23-1
Wavelength (Å)	1.28226
Space group	*P*4_1_22
Cell dimensions	
a, b, c (Å)	49.03, 49.03, 207.98
α, β, γ (°)	90, 90, 90
Resolution (Å)	44.35–2.14 (2.18–2.14)
*R*_merge_	0.092 (1.839)
*I*/σ(*I)*	15.61 (1.19)
CC1/2	0.99 (0.65)
Reflections total	184,241 (17,005)
Reflections unique	14,846 (1,430)
Completeness (%)	99.5 (98.96)
Multiplicity	12.4 (11.9)
**Refinement**	
R_work_	0.1983 (0.3130)
R_free_	0.2456 (0.3644)
No. atoms	
Protein	1,636
Water	33
Ligands	19
B-factors	
Protein	69.97
Water	63.50
Ligands	87.60
RMSDs	
Bond lengths (Å)	0.009
Bond angles (°)	1.04
Ramachandran plot	
Most favored (%)	95.52
Disallowed	0.0

Values in parentheses refer to the highest-resolution shell.

Abbreviations: DENR, density-regulated reinitiation and release factor; ESRF, European Synchrotron Radiation Facility; MCTS1, multiple copies in T-cell lymphoma-1; RMSD, root-mean-square deviation; SAD, single-wavelength anomalous dispersion

Notably, no zinc finger was predicted in DENR. The partial Zn^2+^ coordination site comprises Cys34, Cys37, and Cys44 and is completed *in crystallo* by MCTS1-His58 of a crystallographically related symmetry mate (Fig 1D). We tested whether MCTS1 His58 mediates the binding of MCTS1 to DENR, but this is not the case because HIS-MCTS1[H58A] is still able to pull down DENR (S2A–S2A’ Fig). A mutant form of DENR that cannot bind MCTS1 (DENR[RAA] described in the Results section “DENR-MCTS1 dimerization is required for activity” below) is still able to bind Zn^2+^ (61% of WT levels), indicating that DENR does not require MCTS1 for Zn^2+^ coordination. DENR has a fourth cysteine at position 53 that could complete the Zn^2+^ coordination; however, Cys53 is not present in our crystallization construct. Crystallization trials with a DENR construct including Cys53 (residues 24–55) yielded no crystals. Mutation of Cys53 to alanine, however, had almost no impact on Zn^2+^ binding (S1G Fig). Together, these data suggest DENR coordinates Zn^2+^ mainly via three—rather than four—cysteines, as also observed in other proteins [23,24]. We previously reported the discovery of a de novo missense mutation of DENR Cys37 in an autism spectrum disorder patient [25]. Our structure now reveals that Cys37 is part of this zinc finger. Interestingly, this single mutation is sufficient to abolish Zn^2+^ binding by the DENR–MCTS1 complex (S1G Fig). This mutation strongly impairs DENR function [25], suggesting that zinc conjugation is necessary for proper function of the DENR–MCTS1 complex. These cysteine residues in DENR are not conserved in eIF2D, suggesting the coordination of Zn^2+^ is specific to the DENR–MCTS1 complex.

### DENR–MCTS1 dimerization is required for activity

**Fig 2:**
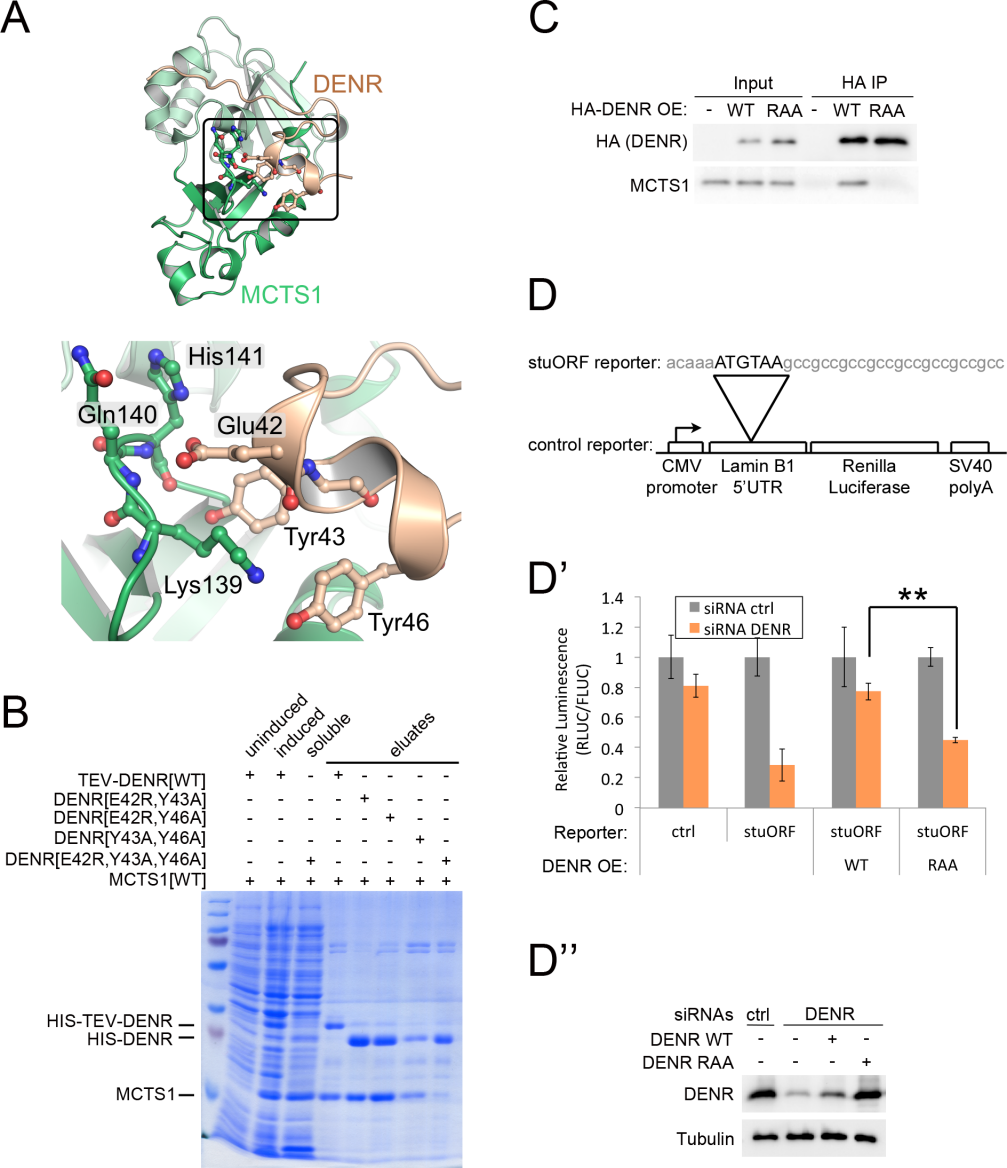
DENR–MCTS1 binding is required for function of the complex. (A) Residues involved in the interaction between DENR and MCTS1. DENR-Glu42 interacts with the nitrogen of the main chain of MCTS1-Gln140 and His141. Furthermore, DENR-Tyr43 and Tyr46 hydrogen-bond with MCTS1-His141 and Lys139, respectively. (B) Mutation of DENR E42, Y43, and Y46—but not any two of these three—abolishes binding to MCTS1 in *E*. *coli*. HIS-tagged DENR variants were coexpressed in *E*. *coli* and purified by affinity chromatography to detect copurifying MCTS1 (representative of 4 biological replicates). (C) Triple DENR mutation E42R, Y43A, Y46A (“RAA”) abolishes binding of DENR to MCTS1 in HeLa cells. HeLa cells were transfected to express DENR[WT] (“WT”), DENR[RAA] (“RAA”), or neither (“-“). DENR–MCTS1 binding was assayed by immunoprecipitating HA-tagged DENR variants and detecting coprecipitating endogenous MCTS1 by immunoblot representative of 2 biological replicates). (D-D') DENR must bind MCTS1 to be functionally active. (D-D”) Activity of DENR[E42R, Y43A, Y46A] (“RAA”) assayed by reconstituting DENR-knockdown HeLa cells by reexpressing (“OE”) either WT or mutant DENR. DENR[WT] and DENR[RAA] contain synonymous substitutions to avoid siRNA-mediated knockdown. Activity is assayed as the ability to promote translation reinitiation downstream of a stuORF as previously reported [10] (*n* = 3). (D”) Since DENR[RAA] is less stable than DENR[WT] (see S2D Fig), the amount of DENR[RAA] overexpression plasmid was elevated to yield at least as much protein as DENR[WT]. Shown here are DENR levels from the same set of cells as in (D’). Underlying data available in S1 Data. CMV, cytomegalovirus; DENR, density-regulated reinitiation and release factor; FLUC, firefly luciferase; HA, human influenza hemagglutinin; HIS, polyhistidine; IP, immunoprecipitated; MCTS1, multiple copies in T-cell lymphoma-1; RLUC, Renilla luciferase; siRNA, small interfering RNA; stuORF, upstream open reading frame with a strong initiation context; SV40, simian virus 40; TEV, tobacco etch virus; WT, wild-type

The N-terminus of DENR (residues 25–33) is unfolded and crawls along the N-terminal domain of unknown function 1947 (DUF1947) of MCTS1 (Fig 2A). The majority of residues involved in DENR binding are conserved—in particular, Leu82, Leu170, and Trp175 (S2B Fig), which form a hydrophobic surface. DENR mostly interacts with MCTS1 via the main chain, except DENR-Tyr33, which hydrogen-bonds with MCTS1-His86. An extensive network of hydrogen bonds is formed between DENR-Glu42 and the backbones of MCTS1-Gln140 and His141, between DENR-Tyr43 and MCTS1-Lys139, between DENR-Tyr43 and the carbonyl of MCTS-His141, and between DENR-Tyr46 and MCTS1-Lys139. Although MCTS1-Lys139 is highly conserved, Gln140 and His141 are not conserved at all, which is in agreement with the interaction only requiring the peptide backbone (S2B Fig). Overall, the interaction between MCTS1 and the DENR buries a surface of 733.5 Å^2^ and 834.9 Å^2^ corresponding to 7.8% and 33.6% of total solvent accessible surface on the MCTS1 and DENR N-terminal region, respectively.

To assess the contribution of DENR Glu42, Tyr43, and Tyr46 to MCTS1 binding, we generated DENR variants with these three residues mutated either pairwise or all three together (E42R, Y43A, and Y46A; henceforth DENR[RAA]). Although mutating any two of these three residues did not abolish binding between DENR and MCTS1 in *E*. *coli*, mutating all three markedly reduced the ability of DENR and MCTS1 to bind each other (Fig 2B and S2C Fig). DENR[RAA] was also unable to bind MCTS1 in HeLa cells, assayed via coimmunoprecipitation assay (Fig 2C). These data identify this interaction surface as the relevant one for DENR–MCTS1 binding and pinpoints Glu42, Tyr43, and Tyr46 as important residues for this interaction.

We previously used a luciferase reporter bearing a very short upstream open reading frame with a strong initiation context (stuORF) as a readout for the ability of DENR–MCTS1 to promote translation reinitiation in vivo (Fig 2D) [10,17,25]. This revealed that both DENR and MCTS1 are necessary for efficient translation reinitiation. Since DENR and MCTS1 bind each other, we next asked whether the ability to form a heterodimer is important for their function. We depleted HeLa cells of endogenous DENR via small interfering RNAs (siRNAs), we reconstituted the cells with either DENR[WT] or DENR[RAA] using constructs that escape siRNA-mediated knockdown, and then we assayed reinitiation activity using the stuORF construct (Fig 2D–2D''). While performing these reconstitution experiments, we found that DENR[RAA] is less well expressed than DENR[WT] (S2D Fig). Hence, for the reconstitution experiments, we increased the amount of DENR[RAA] expression plasmid compared to the DENR[WT] plasmid so as to have at least as much DENR[RAA] protein as DENR[WT] protein (Fig 2D''). Nonetheless, DENR[RAA] was significantly impaired in its ability to promote translation reinitiation compared to the WT protein: as previously reported, knockdown of DENR causes stuORF reporter activity to drop compared to a control reporter lacking the stuORF (second group of bars, Fig 2D'). Reexpression of DENR[WT] restores stuORF reporter activity (third set of bars, Fig 2D'). In contrast, DENR[RAA] is impaired in promoting stuORF reporter activity (fourth set of bars, Fig 2D'), indicating that heterodimerization of the DENR–MCTS1 complex is important for its activity in translation reinitiation.

### DENR–MCTS1 binds tRNA in vitro in the absence of ribosomes

MCTS1 contains a PUA domain, which is frequently found in tRNA binding proteins [26]. Indeed, the related protein eIF2D is able to promote Met-tRNA_i_^Met^ recruitment to the ribosomal 40S subunit [6,7]. Furthermore, DENR and MCTS1 are predicted to be in close proximity to tRNA when the structures of DENR and MCTS1 bound to the ribosomal 40S subunit are superposed with the structure of the PIC containing tRNA_i_^Met^ [20]. To our knowledge, direct binding of DENR–MCTS1 to tRNA, however, has not yet been demonstrated in vitro. To study whether DENR–MCTS1 binds tRNA, we purified recombinant full-length DENR, MCTS1, or the coexpressed DENR–MCTS1 complex from *E*. *coli* and performed gel shift assays with yeast tRNA (S3A Fig). Unlike MCTS1 alone or DENR alone, which showed little or no tRNA binding ability, the DENR–MCTS1 complex was able to significantly retard the mobility of tRNA (S3A Fig). Consistent with DENR and MCTS1 needing to heterodimerize to bind tRNA, the DENR[RAA] mutant, which is not able to bind MCTS1, also did not shift tRNA (S3B Fig). Interestingly, we observed significantly blunted tRNA binding capacity when MCTS1 and DENR were purified individually from bacteria and then mixed together in vitro (“DENR+MCTS1,” S3A Fig), raising the possibility that the complex needs to form in vivo to be functional. The binding of DENR–MCTS1 to tRNA was not strongly influenced by salt concentrations up to 500 mM (S3C Fig). We next asked if the DENR–MCTS1 complex has differential binding affinity for different tRNAs. Binding assays with three different yeast tRNAs revealed that the DENR–MCTS1 complex binds iMet-tRNA and Lys-tRNA more readily than Cys-tRNA and that tRNA binding is not affected by the state of tRNA acylation (S3D Fig). We also assayed binding to in vitro–transcribed human tRNAs (S3E Fig) and found that DENR–MCTS1 binds all the tested tRNAs with similar affinity (S3E’ Fig). This is similar to what was described for eIF2D [6], indicating either that these reinitiation factors are less proficient than eIF2 at discriminating between tRNAs or that additional factors may be involved in vivo to improve tRNA selectivity.

**Fig 3:**
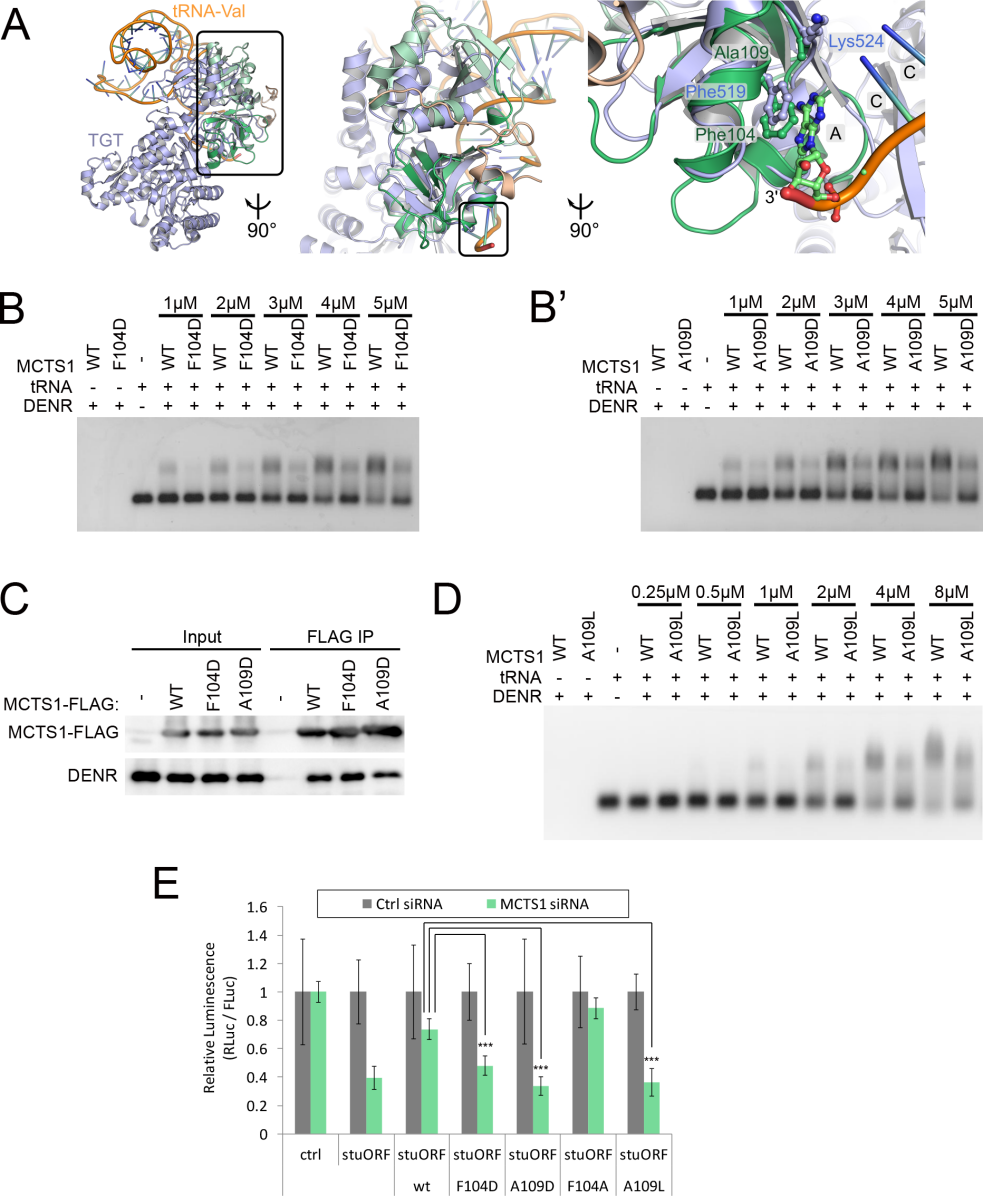
Binding of the DENR–MCTS1 complex to tRNA is required for function. (A) Comparison of MCTS1 with a TGT (PDB-ID: 1J2B). The TGT (light blue, only one molecule from dimer shown) also contains a PUF1947 and PUA domain that directly interact with the tRNA (ribbon, orange). Although the C-terminal MCTS1 domain superimposes well, the N-terminal PUF1947 domains exhibit differences. MCTS1-Phe104 and TGT-Phe519 stack against the last base in the acceptor stem, thereby “measuring” the length of the tRNA. At position of MCTS1-Ala109, TGT contains a lysine residue. (B-B') MCTS1 mutations F104D and A109D strongly impair tRNA binding, assayed by gel shift assay representative of 4 biological replicates). (C) MCTS1 mutations F104D and A109D do not impair binding to DENR. FLAG-tagged MCTS1 variants were IP from HeLa cells, and coimmunoprecipitating endogenous DENR was detected by immunoblot (representative of 2 biological replicates, except the mild drop in DENR binding by the A109D mutation, which is not representative—see S4A Fig). (D) MCTS1 mutation A109L impairs tRNA binding, assayed by gel shift assay (representative of 3 biological replicates). (E) The MCTS1 F104D, A109D, and A109L mutations impair the ability of the DENR–MCTS1 complex to promote translation reinitiation, whereas the F104A mutation does not. Activity of MCTS1 mutants is assayed by reconstituting MCTS1-knockdown HeLa cells with mutated MCTS1 overexpression constructs. Overexpression constructs also contain synonymous substitutions to avoid siRNA-mediated knockdown. Activity is assayed as the ability to promote translation reinitiation downstream of a stuORF as previously reported in [10]. (*n* = 4). Underlying data available in S1 Data. DENR, density-regulated reinitiation and release factor; FLuc, firefly luciferase; IP, immunoprecipitated; MCTS1, multiple copies in T-cell lymphoma-1; PUA, pseudouridine synthase and archaeosine transglycosylase; PUF1947; RLuc, Renilla luciferase; siRNA, small interfering RNA; stuORF, upstream open reading frame with a strong initiation context; TGT, tRNA-guanine transglycosylase; WT, wild type.

To predict residues in the MCTS1 PUA domain contributing to tRNA binding, we superposed MCTS1 on the archeal tRNA-guanine transglycosylase (TGT) in complex with a tRNA [27]. The TGT contains a PUA domain, which was used for structural alignment (Fig 3A), leading to the prediction that MCTS1 Phe104 might be involved in tRNA binding. MCTS1 Phe104 resides at a position similar to a phenylalanine in the TGT-PUA domain, which stacks against the last base of the tRNA acceptor arm (Phe519, Fig 3A, right panel). In contrast, MCTS1 Ala109 is positioned in a location where a bulkier residue would interfere with tRNA binding. To test these predictions, we performed gel shift assays and found that mutating either Phe104 or Ala109 to a bulkier aspartate leads to significantly less tRNA binding by the DENR–MCTS1 complex (Fig 3B–3B'). Importantly, MCTS1 containing either mutation was still able to coimmunoprecipitate endogenous DENR in HeLa cells (Fig 3C and S4A Fig) and had similar melting curves to WT MCTS1 (S4B Fig), indicating that MCTS1[F104D] and MCTS1[A109D] are properly folded and that these two sites specifically impair tRNA binding but not DENR binding. Mutating MCTS1 Ala109 to Leu, which conserves hydrophobicity, also blunted tRNA binding (Fig 3D) but not DENR binding (S4A Fig). In contrast to the F104D mutation, introducing negative charges at a number of other surface residues such as G100D, R54E, and R74E did not impair tRNA binding (S5B Fig). Together, these data show that the DENR–MCTS1 complex is able to bind tRNA in vitro in the absence of ribosomes.

### Binding of DENR–MCTS1 to tRNA is important for reinitiation function

We next tested whether tRNA binding by the DENR–MCTS1 complex is required to promote translation reinitiation in HeLa cells. We used the stuORF reporter assay and a reconstitution setup whereby we knocked down endogenous MCTS1 with siRNAs and then transfected the cells to reexpress either WT or mutant MCTS1 (Fig 3E). Unlike WT MCTS1—which fully restored stuORF reporter activity in MCTS1-knockdown cells—MCTS1[F104D], MCTS1[A109D], and MCTS1[A109L] were impaired in their ability to promote reinitiation (Fig 3E and S5C–S5C’ Fig). Unlike the DENR dimerization mutant, MCTS1[F104D] and MCTS1[A109D] were both expressed at equally high levels as WT MCTS1 (S5C’ Fig), excluding this as a possible explanation for their impaired function and showing that tRNA binding does not affect stability of the DENR–MCTS1 complex. Mutating MCTS1 Phe104 to alanine impaired neither tRNA binding (S5A Fig) nor reinitiation activity (Fig 3E), indicating that this mutation is milder than the F104D mutation and that tRNA binding and reinitiation activity correlate. Altogether, these data indicate that the ability of the DENR–MCTS1 complex to bind tRNA is critical for its ability to promote translation reinitiation, thereby identifying a molecular function for this complex required for its activity.

## Discussion

**Fig 4:**
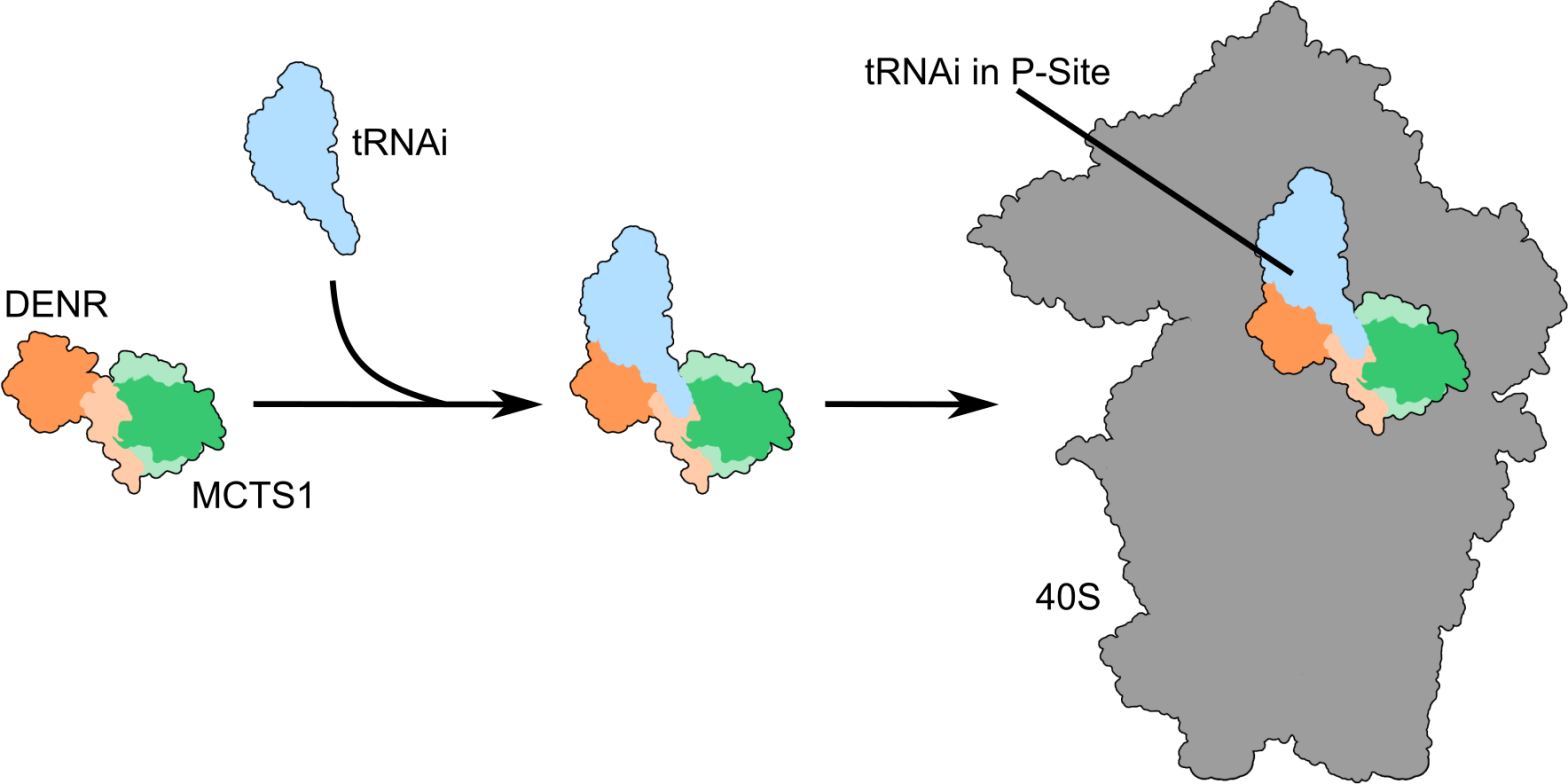
Putative model for the role of DENR–MCTS1 in translation initiation. DENR–MCTS1 binds tRNAi and subsequently recruits it to the 40S ribosomal subunit for reinitiation. DENR, density-regulated reinitiation and release factor; MCTS1, multiple copies in T-cell lymphoma-1; tRNAi, tRNA interference.

Like translation initiation, translation reinitiation is a succession of carefully orchestrated molecular events. Although this succession of events has been extensively studied for canonical cap-dependent initiation, much less is known about the steps of translation reinitiation. Furthermore, we also do not fully understand which molecular functions are required for the various steps of reinitiation. We present here the crystal structure of MCTS1 bound to a fragment of DENR. Based on this structure, we identify and experimentally validate DENR residues Glu42, Tyr43, and Tyr46 to be important for MCTS1 binding and MCTS1 residue Phe104 to be important for tRNA binding. By mutating these residues, we find that both DENR–MCTS1 heterodimerization and tRNA binding are important molecular functions for this complex to promote translation reinitiation. This thereby links molecular functions of DENR and MCTS1 to their contribution in the translation reinitiation process. We also find that the DENR–MCTS1 complex is able to bind tRNA in the absence of the ribosome, yielding a trimeric complex in vitro. This activity was not detected in the past, most likely because DENR and MCTS1 need to be coexpressed and copurified to be active (see next paragraph). These results raise the possibility that the DENR–MCTS1 complex first binds tRNA in the cytosol and then recruits it to the ribosome, analogous to recruitment of initiator tRNA to the ribosome by eIF2 during canonical initiation (Fig 4).

One unexpected finding was that if we mixed in vitro DENR and MCTS1 proteins that had each been individually expressed and purified in *E*. *coli*, they do form a complex but do not yield a complex capable of binding tRNA (S3A Fig). In contrast, the DENR–MCTS1 complex formed in vivo (when coexpressed and purified out of *E*. *coli* as one complex) readily binds tRNA as indicated by gel shift assays (S3A Fig). One possibility is that one or both of the proteins may require chaperones for optimal complex formation. This finding is likely useful for future work aiming to reconstitute DENR–MCTS1–dependent translation in vitro.

From our structure of MCTS1–DENR(aa24–51), Phe104 of MCTS1 is predicted to stack against the last base in the acceptor stem of tRNA, thereby “measuring” the length of the tRNA and allowing RNAs that terminate at that position to bind efficiently. This may discriminate between tRNAs and other RNA species binding to this site. To promote translation reinitiation, one might predict preferential binding of the DENR–MCTS1 complex to initiator tRNA. Our gel shift assays, however, indicate the DENR–MCTS1 complex binds similarly to all tRNAs in vitro. A similar lack of ability to strongly discriminate between different tRNAs, or between uncharged and acylated tRNAs, was also observed for the homologous protein eIF2D [6]. This broader tRNA-binding capacity may therefore represent a feature of translation reinitiation that is different from canonical cap-dependent translation initiation, or it may indicate that additional factors play a role in vivo in helping DENR–MCTS1 select the right tRNA. Interestingly, the K_d_ we measured for tRNA binding (1.5 μM) is roughly equal to the intracellular concentration of tRNAs in HeLa cells. Future work will be required to dissect these aspects in more detail.

Interestingly, we find that the DENR[RAA] mutant, which has impaired MCTS1 binding, does not express as well in HeLa cells as DENR[WT]. One possibility is that DENR[RAA] has lower protein stability compared to DENR[WT]. This would be in agreement with our previous findings that DENR and MCTS1 are codependent on each other for stability; knocking down either DENR or MCTS1 using multiple different independent siRNAs, either in human cells or in fly cells, causes the other protein to also drop in abundance [10,17,25]. Hence, DENR and MCTS1 may need to form a complex to be stabilized, which would explain the reduced stability of the DENR[RAA] protein.

While drafting this manuscript, a low-resolution crystal structure of DENR and MCTS1 bound to the 40S ribosomal subunit was published [20], as was a low-resolution EM structure of eIF2D bound to the 40S ribosomal subunit [19]. Although binding of DENR and MCTS1 to the 40S was also shown and described in [19], the EM data of this complex are not deposited, and therefore a detailed comparison is not possible. Instead, for [19], we compare our DENR–MCTS1 structure to the structure of the homologous eIF2D, given that DENR and MCTS1 occupy the same binding sites on the ribosome as the corresponding homology regions of eIF2D. Both structures reveal that the suppressors of initiation codon mutations 1 (SUI1) domain of DENR has a similar fold as eIF1 and that DENR binds the ribosome at a similar site as eIF1. The two studies differ, however, in terms of whether DENR and MCTS1 directly touch each other. While a direct contact between both proteins is observed in the 6-Å crystal structure [20], in the 10-Å cryo-EM structure they seem not to interact [19]. How does our X-ray structure of the isolated MCTS1–DENR complex relate to these structures? The N-terminal region of DENR in our heterodimer structure nicely fits to the corresponding density present in the 40S crystal structure [20] (S1H Fig), suggesting that the heterodimer containing the N-terminal region of DENR behaves as a rigid body. Although both the structure of DENR–MCTS1 and of eIF2D bound to the 40S show that these proteins contact tRNA, their conformations and their tRNA contact sites are different. Modeling our DENR–MCTS1 structure onto the eIF2D–40S structure of [19] predicts that MCTS1 Phe104 interacts with tRNA, in agreement with what we observe in our structure and experimentally by gel shift assays. In contrast, the structure of 40S–DENR–MCTS1 [20], when modeled onto the 48S PIC [28], does not predict Phe104 to be in contact with tRNA. Furthermore, residues of MCTS1 such as K139 that are predicted from modeling in [20] to bind tRNA are actually involved in DENR binding in our structure. Hence, the structure in [20] may represent a different step in the reinitiation process.

In sum, our structure of the DENR–MCTS1 complex extends the current understanding of noncanonical translation initiation by defining two distinct functions of this complex at a molecular level. We identify residues on DENR and MCTS1 important for heterodimerization and for tRNA binding and add to the current model of how the DENR–MCTS1 complex promotes translation reinitiation by discovering that this complex can bind tRNA in the absence of the ribosome. Additional work will be needed to refine this model further.

## Materials and methods

### Plasmids and primers

Sequences for all oligos used for clonings are provided in S1 and S2 Tables.

The hDENR ORF was amplified from HeLa cell cDNA using oligos OSS 366/367 and cloned into the NcoI/NotI sites of pET-hisTEV (pETM10) and pET-his vectors. Plasmids for simultaneous expression of MCTS1 and N-terminally HIS-tagged DENR fragments in *E*. *coli* were generated by PCR-amplifying DENR fragments from pET-HIS-DENR, using oligos listed in S1 Table and cloning them into the XbaI/NotI sites of the pETDuet dual-expression vector under the control of a T7 promotor. The human MCTS1 ORF was amplified by PCR with oligos OSS 341/373 and cloned into the MfeI/XhoI sites downstream of the DENR ORF and under the control of its own T7 promotor. All plasmids were verified by sequencing.

To generate double or triple mutations of DENR on aas E42, Y43, and Y46, primers containing combinations of either double or all three mutations together (S1 Table) were used for point mutagenesis in combination with flanking primers OSS 649/367, and the PCR product was cloned directly into the pETDuet-MCTS1-HIS-FL vector using the XbaI/NotI sites. All plasmids were verified by sequencing.

To mutate C-terminally HIS-tagged MCTS1 in the pETDuet vector, upstream and downstream oligos OSS341 and OSS459 were used in combination with primers containing mutations for F104D and A109D (S1 Table) to amplify the mutated ORF, which was cloned into the TOPO vector (Invitrogen), sequenced, and then subcloned in frame with C-terminal 6xHIS into the MfeI/XhoI sites of the pETDuet-DENR vector.

To express N-terminally tagged HA-DENR[WT] and HA-DENR[E42R, Y43A, Y46A] (“HA-DENR[RAA]”) under the control of a CMV promotor in HeLa cells, WT and mutant DENR ORFs were amplified using oligos OSS 072 and OSS 367 from the corresponding bacterial expression plasmids, cloned via EcoRI and NotI sites into a pCDNA-HA expression vector, and sequenced for correctness.

For the DENR and MCTS1 reconstitution luciferase experiments in HeLa cells, firefly luciferase and Renilla luciferase reporters were described in [17]. To express DENR and MCTS1 variants that escape siRNA-mediated knockdown, DENR and MCTS1 ORFs containing synonymous mutations previously described in [17] were used as templates for site-directed mutagenesis using oligos OSS684/OSS685 (for DENR) or OSS702/OSS703 and OSS706/OSS707 (for MCTS1).

To generate C-terminally Flag-tagged MCTS1 as WT, F104D, and A109D versions, WT and mutant MCTS1 ORFs were amplified with primers OKE084 and OSS 745 (containing a FLAG ORF, full sequences in S2 Table) from the bacterial expression vectors, cloned into pRK containing a CMV promoter, and sequenced for correctness.

### Antibodies

Antihuman DENR and antihuman MCTS1 for immunoblotting were raised in the lab by immunizing guinea pigs with full-length recombinant proteins. Anti tubulin was purchased from Sigma (T9026).

### Protein expression and purification

Proteins were expressed using *E*. *coli* BL21 (DE3) cells in 2YT media supplemented with either Kanamycin (30 μg/ml) or Ampicillin (100 μg/ml), depending on the plasmid used. Cells were grown to an OD_600_ of 0.8–1.0 at 37°C, then shifted to 18°C. Expression was induced with the addition of 0.4 mM IPTG, and cells were grown further overnight, harvested by centrifugation, and the cell pellets either used immediately for lysis and purification or frozen with LN_2_ and stored at −20°C.

All variants of the DENR–MCTS1 complex were purified via an N- or C-terminal His_6_-tag using NiNTA and SEC. Cells were resuspended in lysis buffer (30 mM HEPES, 30 mM Imidazol, 500 mM NaCl) and lysed with a Microfluidizer (Microfluidics) at 0.55 MPa. The lysate was cleared by centrifugation for 35 min at 35,000 × g and 4°C, and the resulting supernatant was applied to a 2 ml NiNTA column. The column was washed with 25–50 column volumes of lysis buffer and eluted with elution buffer (lysis buffer plus 400 mM Imidazol). The NiNTA-eluate was applied to a Superdex 200 26/60 column, equilibrated with SEC-buffer I (10 mM HEPES pH 7.5, 500 mM NaCl). Peak fractions containing the DENR–MCTS1 complex were pooled, concentrated to 10–15 mg/ml, and either used directly or shock-frozen with LN_2_ and stored at −80°C.

### Crystallization and structure determination

Attempts to crystallize the full-length DENR–MCTS1 complex as well as various truncations were not successful, and therefore we focused on a minimal complex. The minimal DENR–MCTS1 complex was purified as described in the section “Protein expression and purification” above from bacteria coexpressing untagged full-length WT MCTS1 together with NHis_6_-tagged DENR aas 24–51. Crystals were obtained by the sitting drop vapor diffusion method at 18°C in a condition containing 0.2 M AmSO_4_ and 20% PEG3350. Prior to data collection, crystals were harvested in reservoir solution supplemented with 20% glycerol and flash-cooled with liquid nitrogen. Diffraction data were collected at ESRF beamline ID23-2 and integrated with XDS [29] and further processed with AIMLESS [30] from the CCP4 package [31]. The crystals belong to the space group *P*4_1_22. The structure was solved by molecular replacement as implemented in MOLREP [32]. Coordinates of the MCTS1 crystallization variant (PDB-ID: 3R90) were used as a search model. Very strong positive density was observed near His58. The structure was manually rebuilt in COOT [33] and refined with REFMAC5 [34] and PHENIX [35]. Anomalous density maps were calculated with ANODE [36], showing a clear peak (14 σ) near His58, which was initially assigned to Zn^2+^. The identity of the Zn^2+^-ion was confirmed with an XRF spectrum performed at ESRF beamline ID23-1 [37]. XRF data were analyzed with PyMCA [38]. Based on the knowledge that a Zn^2+^ ion is bound between the two proteins, we collected one dataset on the Zn-edge and were also able to solve the structure de novo by means of Zn-SAD. Data preparation, heavy atom location, and phasing were performed with SHELXC/D/E [39] pipeline navigated with HKL2MAP [40]. The final structure contains one DENR–MCTS1 complex. Data collection and refinement statistics are summarized in Table 1. Structural comparisons were performed with GESAMT [41]. Determination of conserved residues and projection on the surface was performed with ConSurf [42]. All structural figures were prepared with PyMOL.

### Coimmunoprecipitation experiments in HeLa cells

For coimunoprecipitation experiments, HeLa cells were seeded overnight and transfected the next day with expression constructs using Effectene (Qiagen) according to manufacturer’s protocol. After 2 d of expression, cells where harvested and lysed in RIPA buffer (50mM Tris, pH 7,5, 150 mM NaCl, 1% Sodiumdesoxycholate, 0,1% SDS, 1% Nonidet P-40) supplemented with protease inhibitors (Roche) and benzonase (Merck). After spinning for 10 min at 10,000 g to remove unsolubilized cell debris, prewashed HA-Agarose Affinity Matrix (Roche 11815016001, used in Fig 2C) or anti-Flag Affinity Beads (Sigma A2220, used in Fig 3D) were added to the suspension. After 1.5 h of rotation in the cold room, the beads where collected by centrifugation, washed with RIPA buffer 4 times, and subjected to PAGE, followed by immunoblotting.

### Reconstitution assays in HeLa cells

For reconstitution experiments, HeLa cells were reverse transfected with control siRNA (anti lacZ, Dharmacon #D00-2000-01-20) or siRNAs against DENR or MCTS1 (Dharmacon, sequences provided in S3 Table), using RNAi Max (Thermo Scientific). Samples in triplicate in 96-well format for luciferase assays were treated in parallel with western blot samples in 24-well format. After 2 d of knockdown, all samples where transfected with luciferase reporters, with or without rescue constructs, as published in [17], and incubated for 2 d longer. Plasmid DNA amounts transfected per 24-well were as follows: 0.5 μg of each FLuc and Rluc reporter plasmid; and for reconstituting DENR levels, 0.1 μg of pCDNA-DENR[WT] or 0.26 μg of pCDNA-DENR[RAA] plasmids. The cells for immunoblotting were then lysed in RIPA buffer containing protease inhibitors and benzonase for 5 min at room temperature. Lysates were then clarified by centrifugation for 5 min at 10,000 g. Finally, Laemmli buffer was added to 1x final concentration for SDS-PAGE and immunoblotting. For luciferase assays, cells were lysed and analyzed with the Dual-Luciferase Reporter Assay System from Promega (#E1960).

### tRNAs

Yeast tRNA mix was purchased from Ambion (#AM7119). Individual yeast tRNAs, either acylated or nonacylated, were purchased from tRNA Probes, College Station, USA (iMet tRNA cat# MI-03; iMet tRNA-Met cat# MI-60; Lys tRNA cat# L-03; Lys tRNA-Lys cat# L-60; Cys tRNA cat# C-03; Cys tRNA-Cys cat# C-60).

In vitro–transcribed human tRNAs were produced by annealing oligonucleotides containing a 5′ T7 promoter followed by the tRNA sequence. Oligo sequences are provided in S4 Table. The oligos were annealed by heating up to 95°C and slowly equilibrating to room temperature. tRNAs were in vitro–transcribed by combining and incubating annealed oligos (1 μM), NTPs (2 mM), Inorganic Phosphatase (3 U/100 μL), Ribolock (100U/100 μL), and T7-RNA Polymerase (60U/100 μL; all from Thermo Scientific) for 3 h at 37°C.

After in vitro transcription reactions, all RNAs were purified using Trizol (Thermo Scientific), following manufacturer’s instructions.

### tRNA gel shift assays

For tRNA electrophoretic mobility shift assays, either single HIS-tagged DENR or MCTS1 was expressed in *E*. *coli* Rosetta pLysS cell and purified by His-Trap affinity columns (GE Healthcare), or the two proteins were coexpressed and purified by Ni-NTA Agarose beads (Qiagen). Proteins were eluted in reaction buffer (50 mM Tris pH 7.5, 150 mM NaCl, 15 mM Imidazole, Roche Protease inhibitors without EDTA) containing 300 mM imidazole. After elution, 2 mM DTT was added. Varying amounts of protein were mixed together with RNA and 0.5 μl Ribolock (Thermo Scientific #EO0381) in a total volume of 20 μL of reaction buffer to obtain final protein concentrations of 0.25 μM to 16 μM in the reaction mix. Final RNA concentrations in the binding reactions were 3 μM for the yeast tRNA mix and otherwise 1 μM. After 30 min of incubation on ice, loading buffer containing DNA/RNA dye GelRed was added to the samples and loaded on a native agarose gel, followed by electrophoresis at 9 volts/cm of gel length for 33 min at 4°C. For calculating K_d_’s for tRNA binding, the integrated density of the shifted band was quantified using ImageJ. From this, the percentage of tRNA bound to DENR•MCTS1 (“theta”) was calculated for each DENR–MCTS1 concentration. This was used to derive the amount of tRNA•DENR•MCTS1 complex and the amount of free DENR•MCTS1. The linear regression on a Scatchard was then used to calculate the Kd. This was done for 5 biological replicates.

### Quantification of Zn^2+^ binding

Zn^2+^ binding was assayed using a protocol adapted from [21,22]: DENR–MCTS1 proteins were expressed in *E*. *coli* and purified in the absence of DTT as described above in the section “Protein expression and purification.” After elution in elution buffer (20 mM Hepes pH7.5, 150 mM NaCl, 330 mM Imidazole), both WT and mutant proteins were diluted in elution buffer to an equal concentration of 1.1 mg/ml. Protein solutions were then treated with 10 mM NEM (N-Ethylmaleimide, Sigma, #E3876) for 10 min at 37°C to release the Zn^2+^. The samples were then heated for 5 min at 95°C to completely denature the proteins and then centrifuged for 5 min at 20,000 g to spin down the precipitates. Zinc was then measured from the supernatant. To measure zinc, 40 μl of 5 mM PAR (Sigma #323209) was combined with 15 μl of ZnCl_2_ standard solutions (from 1 μM to 100 μM, also in elution buffer) or with 15 μl of cleared sample solution. The samples were mixed, incubated at room temperature for 5 min, and absorption at 500 nm was then measured.

### Melting curves

Melting temperatures of wild-type and mutant DENR–MCTS1 complexes were determined with a Prometheus NT.48 (Nanotemper, Germany) at concentrations of 1 mg/ml. A temperature gradient from 20–80°C with a speed of 1.5°C/min was run while tryptophan fluorescence at 330 and 350 nm was recorded. Melting temperatures were determined using the manufacturer-supplied software, which calculates the ratio between fluorescence counts at 350 and 330 nm. The 350/330 ratio is plotted against temperature, and the first derivative is used to determine the melting points.

## Supporting information

S1 Fig Support to main Fig 1. **(A)** The first 46 aas of DENR are required for MCTS1 binding. HIS-tagged MCTS1 was coexpressed with WT or mutant DENR lacking the first 46 aas (DENR-47-C) in *E*. *coli*, and binding was assessed by purifying MCTS1 via nickel affinity purification and detecting copurifying DENR. − uninduced, + induced with IPTG, “P” (insoluble pellet), “SN” (soluble supernatant), “FT” (flow-through on nickel column), “E” (eluate). **(B-E)** Successive truncations of the N-terminus of DENR identify aas 24–51 as the minimum peptide capable of binding MCTS1. **(F)** XRF emission spectra of a typical DENR–MCTS1 crystal recorded from 2–14 keV at ESRF beamline ID23-1. The peak at 8.639 keV indicates the presence of Zinc. **(G)** Cys37 of DENR is involved in zinc binding. DENR–MCTS1 proteins containing the indicated DENR mutations were purified from *E*. *coli*, and the bound Zn^2+^ was quantified using PAR, which absorbs at 500 nM upon Zn^2+^ binding. “4Cmut” indicates mutation of all four cysteines C34A, C37Y, C44A, and C53A. (*n* = 3, error bars = SD). Underlying data available in S1 Data. **(H)** MCTS1 structure is essentially (RMSD of 0.716 Å for 181 residues) the same as the one previously reported for MCTS1 containing the three mutations E137A, K139A, and Q140A ("MCTS1x"; PDB-ID: 3R90, chain A). **(I)** Docking of the minimal DENR–MCTS1 structure into the electron density map of 5VYC. Electron density for MCTS1 (2Fo-Fc, blue mesh) and DENR-N (Fo-Fc, orange mesh) both contoured at 1.5σ is shown. The DENR peptide does not fully occupy the density, suggesting that more residues form a stable fold than are present in our construct. Lack of continuous density between the DENR-N and -C suggests a flexible linker (dashed lines). aa, amino acid; DENR, density-regulated reinitiation and release factor; ESRF, European Synchrotron Radiation Facility; HIS, polyhistidine; MCTS1, multiple copies in T-cell lymphoma-1; PAR, 4-(2-pyridylazo)resorcinol; RMSD, root-mean-square deviation; WT, wild-type; XRF, X-ray fluorescence.
(TIF)

S2 Fig Support to main Fig 2. **(A)** Mutating MCTS1 His58 to alanine does not abolish binding between MCTS1 and either full-length DENR (A) or the DENR aas 24–51 peptide (A'). DENR and MCTS1 variants were coexpressed in *E*. *coli*. The HIS-tagged protein was affinity purified over a nickel column, and copurification of the partner protein was assessed in the eluate. “FT” (flow-through on nickel column), “W” (wash), “E” (eluate). **(B)** Surface view of MCTS1 with residues colored by conservation (from cyan green to dark red being variable to conserved, respectively). Residues along the DENR binding region are highly conserved, except Gln140 and His141, since interaction does not involve the side chains but the backbone (inset, detailed view in lower panel). **(C)** The triple DENR mutation E42R, Y43A, Y46A, but not the double mutation Y43A, Y46A, abolishes binding of DENR to MCTS1. HIS-tagged DENR variants were expressed in *E*. *coli* together with full-length untagged MCTS1. HIS-tagged DENR was affinity purified over a nickel column, and copurifying MCTS1 was assessed in the eluate. “FT” (flow-through on nickel column), “W” (wash), “E” (eluate). **(D)** DENR[E42R, Y43A, Y46A] ("DENR[RAA]") is less stable or less well expressed than DENR[WT]. Endogenous DENR was knocked down with siRNAs in HeLa cells, and DENR expression was reconstituted by expressing WT or mutant DENR containing synonymous mutations in the ORF to escape siRNA-mediated knockdown. While 0.4 μg of DENR[WT] expression plasmid reconstitutes DENR to endogenous protein levels (lanes 1 and 6), 0.4 μg of DENR[RAA] expression plasmid does not. aa, amino acid; DENR, density-regulated reinitiation and release factor; HIS, polyhistidine; MCTS1, multiple copies in T-cell lymphoma-1; ORF, open reading frame; siRNA, small interfering RNA; WT, wild-type.
(TIF)

S3 Fig Support to main Fig 3. **(A)** The DENR–MCTS1 complex, obtained by coexpressing DENR and MCTS1 in *E*. *coli*, binds tRNA, assayed by gel shift assay using yeast tRNAs. Each protein by itself does not bind tRNA, and the complex reconstituted by expressing and purifying each protein singly out of bacteria and then mixing them in the reaction tube also does not bind tRNA. "DENR–MCTS1" indicates that the two proteins were coexpressed in bacteria, whereas "DENR+MCTS1" indicates each protein was expressed and purified singly in *E*. *coli* and then mixed together. (Representative of 3 biological replicates). **(B)** DENR[E42R, Y43A, Y46A] ("RAA"), which cannot bind MCTS1, does not bind tRNA, assayed by gel shift assay using yeast tRNAs. **(C)** DENR–MCTS1 binds tRNA at a range of salt concentrations, from 150 mM NaCl to 500 mM NaCl. **(D)** Binding of DENR–MCTS1 to yeast tRNAs in their nonacylated (lanes 1–6) or acylated states (lanes 7–12). DENR–MCTS1 binds Cys-tRNA less well than iMet-tRNA or Lys-tRNA and does not discriminate between acylated and nonacylated tRNAs in this in vitro setup. Binding assayed by gel shift assay as in (A). **(E-E’)** The DENR–MCTS1 complex binds to various human tRNAs in vitro with roughly similar affinities. (E) Representative examples (*n* = 5). (E’) Quantification of Kd from 5 biological replicates. DENR, density-regulated reinitiation and release factor; MCTS1, multiple copies in T-cell lymphoma-1.
(TIF)

S4 Fig MCTS1[F104D] and [A109D] bind DENR and display similar melting curves as WT MCTS1. **(A)** MCTS1[A09D] and MCTS1[A109L] bind endogenous DENR. FLAG-tagged WT and mutant MCTS1 were expressed in HeLa cells and immunoprecipitated using the FLAG tag. Immunoprecipitates were probed for binding to endogenous DENR. Amount of coimmunoprecipitated DENR, normalized to the amount of FLAG–MCTS1, was quantified using the ChemiDoc system and is shown below the immunoblots. **(B)** Melting curves of WT (green) and mutant DENR–MCTS1 complexes (F104D and A019D, red and blue, respectively). The ratio between fluorescence counts at 350 and 330 nm is plotted against temperature (upper panel). Inflection points in the 350/330 ratio, which correspond to the melting temperature, are determined by the first derivative plotted against temperature (lower panel). The WT complex unfolds at 63.8°C, the F104D mutant at 65.3°C, and the A109D at 64.0°C. DENR, density-regulated reinitiation factor; MCTS1, multiple copies in T-cell lymphoma-1; WT, wild-type.
(TIF)

S5 Fig Support to main Fig 3. **(A)** The MCTS1 F104A mutation does not impair iMet-tRNA binding. tRNA binding assayed by gel shift assays as in S3 Fig. **(B)** Several mutations introducing negative charges on MCTS1 surface residues—such as R54E, R74E, and G100D—do not impair binding to yeast tRNA assayed by gel shift assays. **(C-C')** MCTS1 must bind tRNA to be functionally active. (C) Activity of MCTS1 tRNA-binding mutants, assayed by reconstituting MCTS1-knockdown HeLa cells with mutated MCTS1 overexpression constructs. Overexpression constructs also contain synonymous substitutions to avoid siRNA-mediated knockdown. Activity is assayed as the ability to promote translation reinitiation downstream of a stuORF as previously reported [10]. (C') MCTS1 protein levels from the same set of cells as in (C). Underlying data available in S1 Data. DENR, density-regulated reinitiation and release factor; MCTS1, multiple copies in T-cell lymphoma-1; siRNA, small interfering RNA; stuORF, upstream open reading frame with a strong initiation context.
(TIF)

S6 Fig Replicates of gel shift assays. (TIF)

S7 Fig Replicates of gel shift assays. (TIF)

S1 Table Oligos used to construct bacterial expression plasmids. (XLSX)

S2 Table Oligos used for constructing plasmids for HeLa transfections. (XLSX)

S3 Table Sequences of siRNAs. siRNA, small interfering RNA.
(XLSX)

S4 Table Oligos used for in vitro transcription of tRNAs. (XLSX)

S1 Data Data underlying Fig 2, Fig 3, and S1 and S5 Figs. (XLSX)

## Abbreviations

aa: amino acid
Cdk4: cyclin-dependent kinase 4
DENR: density-regulated reinitiation and release factor
DUF1947: domain of unknown function 1947
eIF1: eukaryotic initiation factor 1
eIF2: eukaryotic initiation factor 2
eIF2D: eukaryotic initiation factor 2D
eIF3: eukaryotic initiation factor 3
eIF4F: eukaryotic initiation factor 4F
GTP: guanosine triphosphate
HIS: polyhistidine
IRES: internal ribosome entry site
MCTS1: multiple copies in T-cell lymphoma-1
MDM2: mouse double minute 2
ORF: open reading frame
PAR: 4-(2-pyridylazo)resorcinol
PIC: preinitiation complex
PUA: pseudouridine synthase and archaeosine transglycosylase
RMSD: root-mean-square deviation
SAD: single-wavelength anomalous dispersion
siRNA: small interfering RNA
stuORF: upstream open reading frame with a strong initiation context
SUI1: suppressors of initiation codon mutations 1
SV40: simian virus 40
SWIB: *SWItch*/Sucrose Nonfermentable complex B
TGT: tRNA-guanine transglycosylase
uORF: upstream open reading frame
WH: winged helix
WT: wild-type
XRF: X-ray fluorescence
