# Supplementary figures and images for: DENR–MCTS1 heterodimerization and tRNA recruitment are required for translation reinitiation
Source: PLoS Biol. 2018 Jun 11;16(6):e2005160. doi: 10.1371/journal.pbio.2005160 (PMC6013234; doi:10.1371/journal.pbio.2005160)

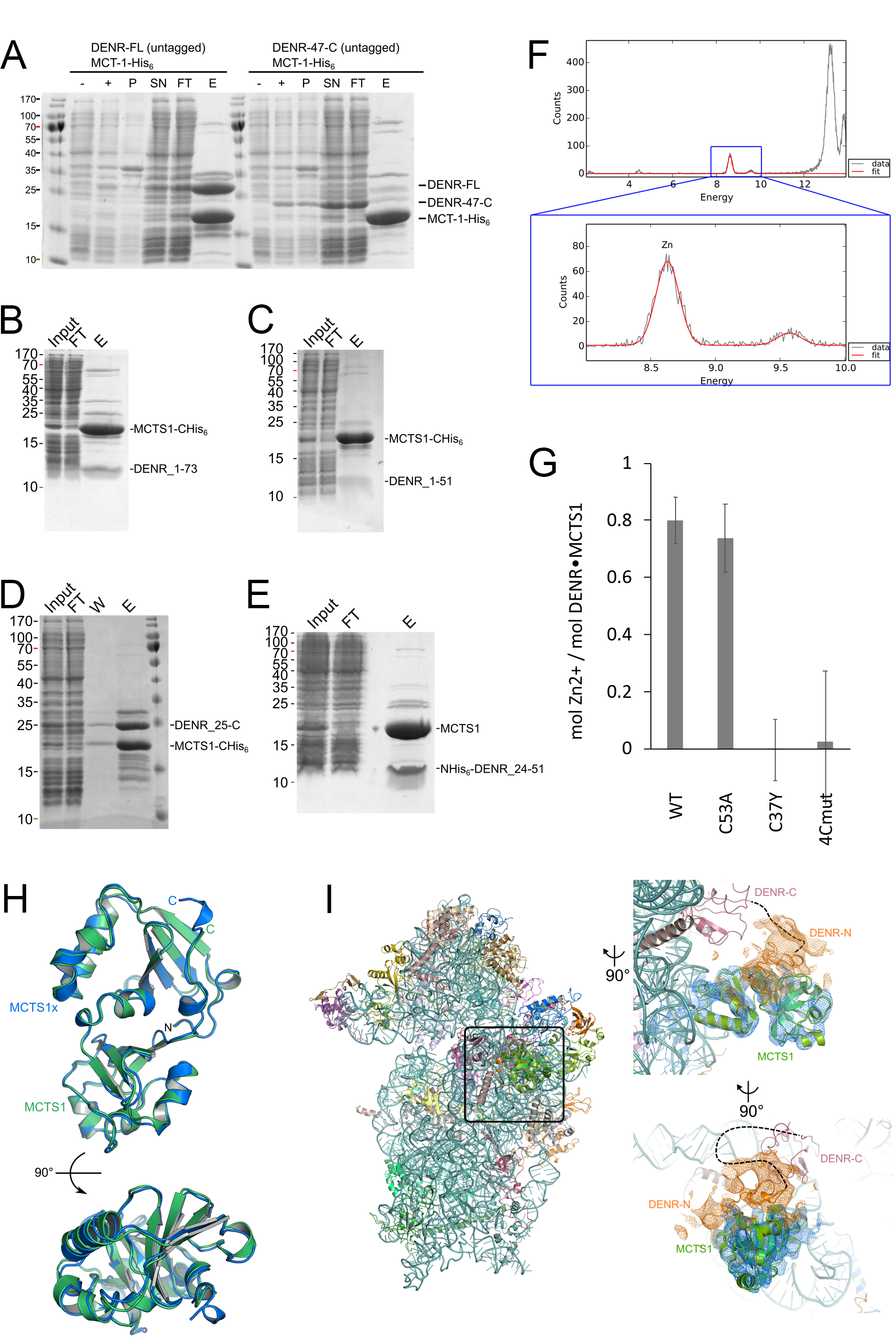

Supplement: S1 Fig — (A) The first 46 aas of DENR are required for MCTS1 binding. HIS-tagged MCTS1 was coexpressed with WT or mutant DENR lacking the first 46 aas (DENR-47-C) in E. coli, and binding was assessed by purifying MCTS1 via nickel affinity purification and detecting copurifying DENR. − uninduced, + induced with IPTG, “P” (insoluble pellet), “SN” (soluble supernatant), “FT” (flow-through on nickel column), “E” (eluate). (B-E) Successive truncations of the N-terminus of DENR identify aas 24–51 as the minimum peptide capable of binding MCTS1. (F) XRF emission spectra of a typical DENR–MCTS1 crystal recorded from 2–14 keV at ESRF beamline ID23-1. The peak at 8.639 keV indicates the presence of Zinc. (G) Cys37 of DENR is involved in zinc binding. DENR–MCTS1 proteins containing the indicated DENR mutations were purified from E. coli, and the bound Zn2+ was quantified using PAR, which absorbs at 500 nM upon Zn2+ binding. “4Cmut” indicates mutation of all four cysteines C34A, C37Y, C44A, and C53A. (n = 3, error bars = SD). Underlying data available in S1 Data. (H) MCTS1 structure is essentially (RMSD of 0.716 Å for 181 residues) the same as the one previously reported for MCTS1 containing the three mutations E137A, K139A, and Q140A ("MCTS1x"; PDB-ID: 3R90, chain A). (I) Docking of the minimal DENR–MCTS1 structure into the electron density map of 5VYC. Electron density for MCTS1 (2Fo-Fc, blue mesh) and DENR-N (Fo-Fc, orange mesh) both contoured at 1.5σ is shown. The DENR peptide does not fully occupy the density, suggesting that more residues form a stable fold than are present in our construct. Lack of continuous density between the DENR-N and -C suggests a flexible linker (dashed lines). aa, amino acid; DENR, density-regulated reinitiation and release factor; ESRF, European Synchrotron Radiation Facility; HIS, polyhistidine; MCTS1, multiple copies in T-cell lymphoma-1; PAR, 4-(2-pyridylazo)resorcinol; RMSD, root-mean-square deviation; WT, wild-type; XRF, X-ray fluorescence. (TIF) [file pbio.2005160.s001.tif]

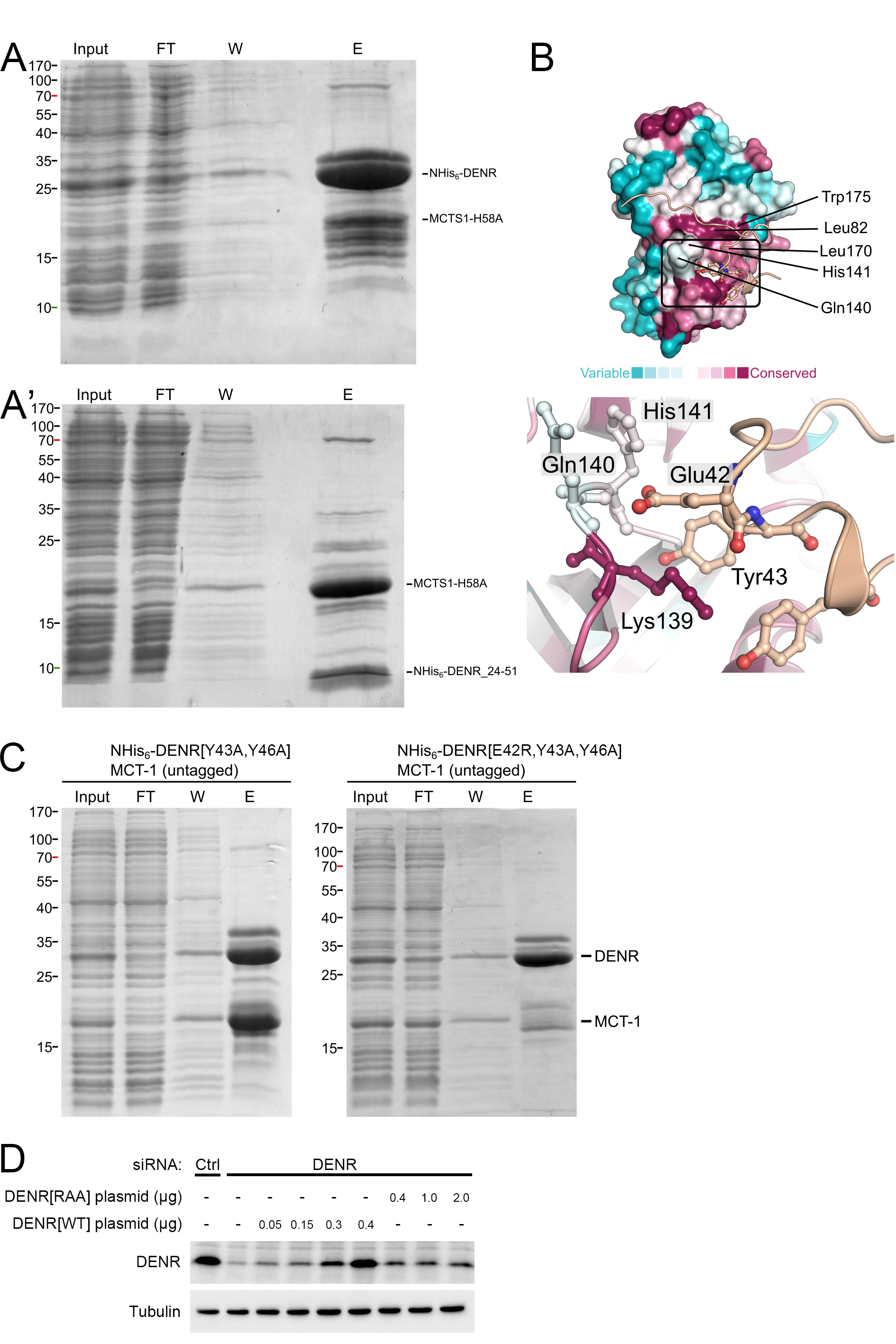

Supplement: S2 Fig — (A) Mutating MCTS1 His58 to alanine does not abolish binding between MCTS1 and either full-length DENR (A) or the DENR aas 24–51 peptide (A'). DENR and MCTS1 variants were coexpressed in E. coli. The HIS-tagged protein was affinity purified over a nickel column, and copurification of the partner protein was assessed in the eluate. “FT” (flow-through on nickel column), “W” (wash), “E” (eluate). (B) Surface view of MCTS1 with residues colored by conservation (from cyan green to dark red being variable to conserved, respectively). Residues along the DENR binding region are highly conserved, except Gln140 and His141, since interaction does not involve the side chains but the backbone (inset, detailed view in lower panel). (C) The triple DENR mutation E42R, Y43A, Y46A, but not the double mutation Y43A, Y46A, abolishes binding of DENR to MCTS1. HIS-tagged DENR variants were expressed in E. coli together with full-length untagged MCTS1. HIS-tagged DENR was affinity purified over a nickel column, and copurifying MCTS1 was assessed in the eluate. “FT” (flow-through on nickel column), “W” (wash), “E” (eluate). (D) DENR[E42R, Y43A, Y46A] ("DENR[RAA]") is less stable or less well expressed than DENR[WT]. Endogenous DENR was knocked down with siRNAs in HeLa cells, and DENR expression was reconstituted by expressing WT or mutant DENR containing synonymous mutations in the ORF to escape siRNA-mediated knockdown. While 0.4 μg of DENR[WT] expression plasmid reconstitutes DENR to endogenous protein levels (lanes 1 and 6), 0.4 μg of DENR[RAA] expression plasmid does not. aa, amino acid; DENR, density-regulated reinitiation and release factor; HIS, polyhistidine; MCTS1, multiple copies in T-cell lymphoma-1; ORF, open reading frame; siRNA, small interfering RNA; WT, wild-type. (TIF) [file pbio.2005160.s002.tif]

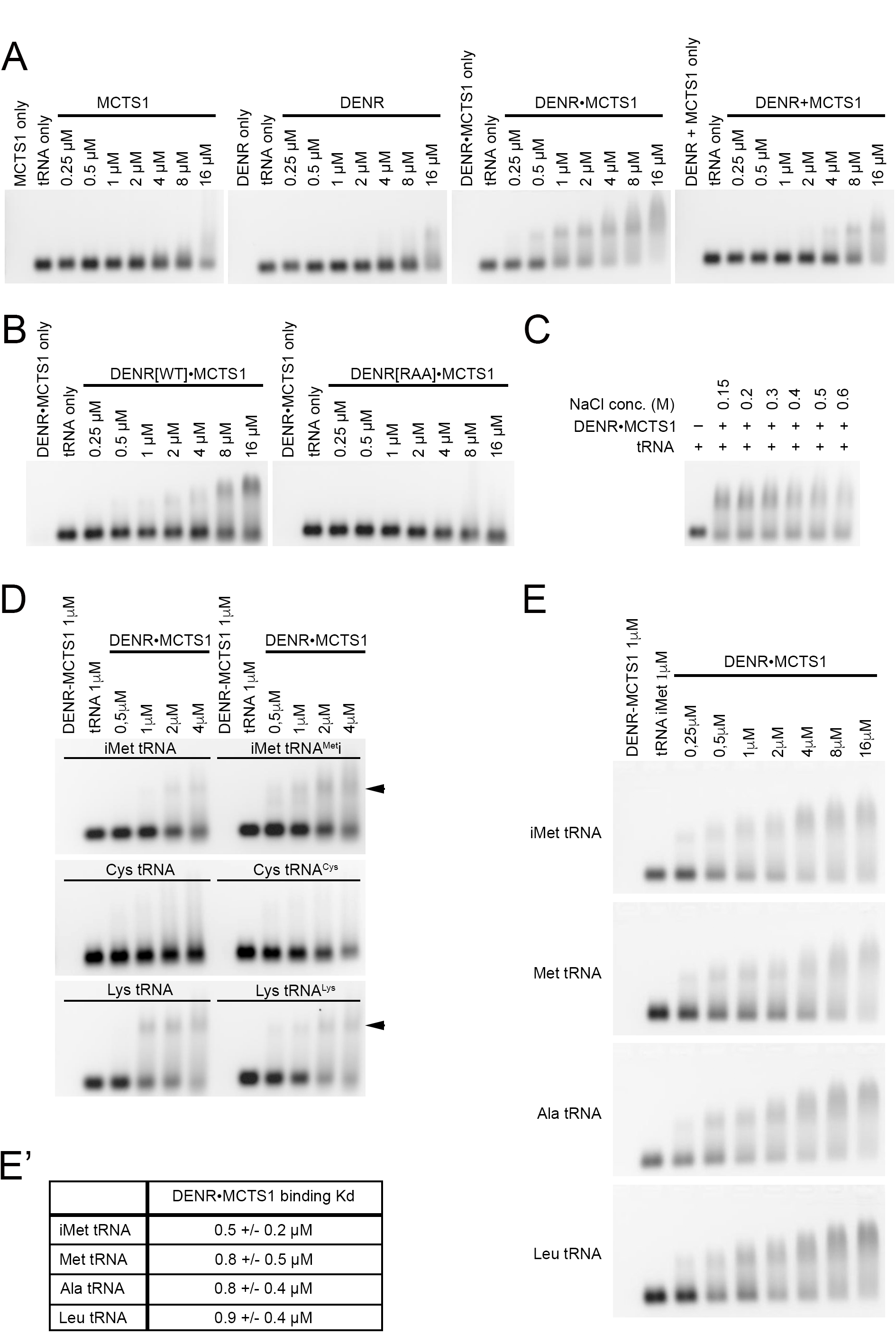

Supplement: S3 Fig — (A) The DENR–MCTS1 complex, obtained by coexpressing DENR and MCTS1 in E. coli, binds tRNA, assayed by gel shift assay using yeast tRNAs. Each protein by itself does not bind tRNA, and the complex reconstituted by expressing and purifying each protein singly out of bacteria and then mixing them in the reaction tube also does not bind tRNA. "DENR–MCTS1" indicates that the two proteins were coexpressed in bacteria, whereas "DENR+MCTS1" indicates each protein was expressed and purified singly in E. coli and then mixed together. (Representative of 3 biological replicates). (B) DENR[E42R, Y43A, Y46A] ("RAA"), which cannot bind MCTS1, does not bind tRNA, assayed by gel shift assay using yeast tRNAs. (C) DENR–MCTS1 binds tRNA at a range of salt concentrations, from 150 mM NaCl to 500 mM NaCl. (D) Binding of DENR–MCTS1 to yeast tRNAs in their nonacylated (lanes 1–6) or acylated states (lanes 7–12). DENR–MCTS1 binds Cys-tRNA less well than iMet-tRNA or Lys-tRNA and does not discriminate between acylated and nonacylated tRNAs in this in vitro setup. Binding assayed by gel shift assay as in (A). (E-E’) The DENR–MCTS1 complex binds to various human tRNAs in vitro with roughly similar affinities. (E) Representative examples (n = 5). (E’) Quantification of Kd from 5 biological replicates. DENR, density-regulated reinitiation and release factor; MCTS1, multiple copies in T-cell lymphoma-1. (TIF) [file pbio.2005160.s003.tif]

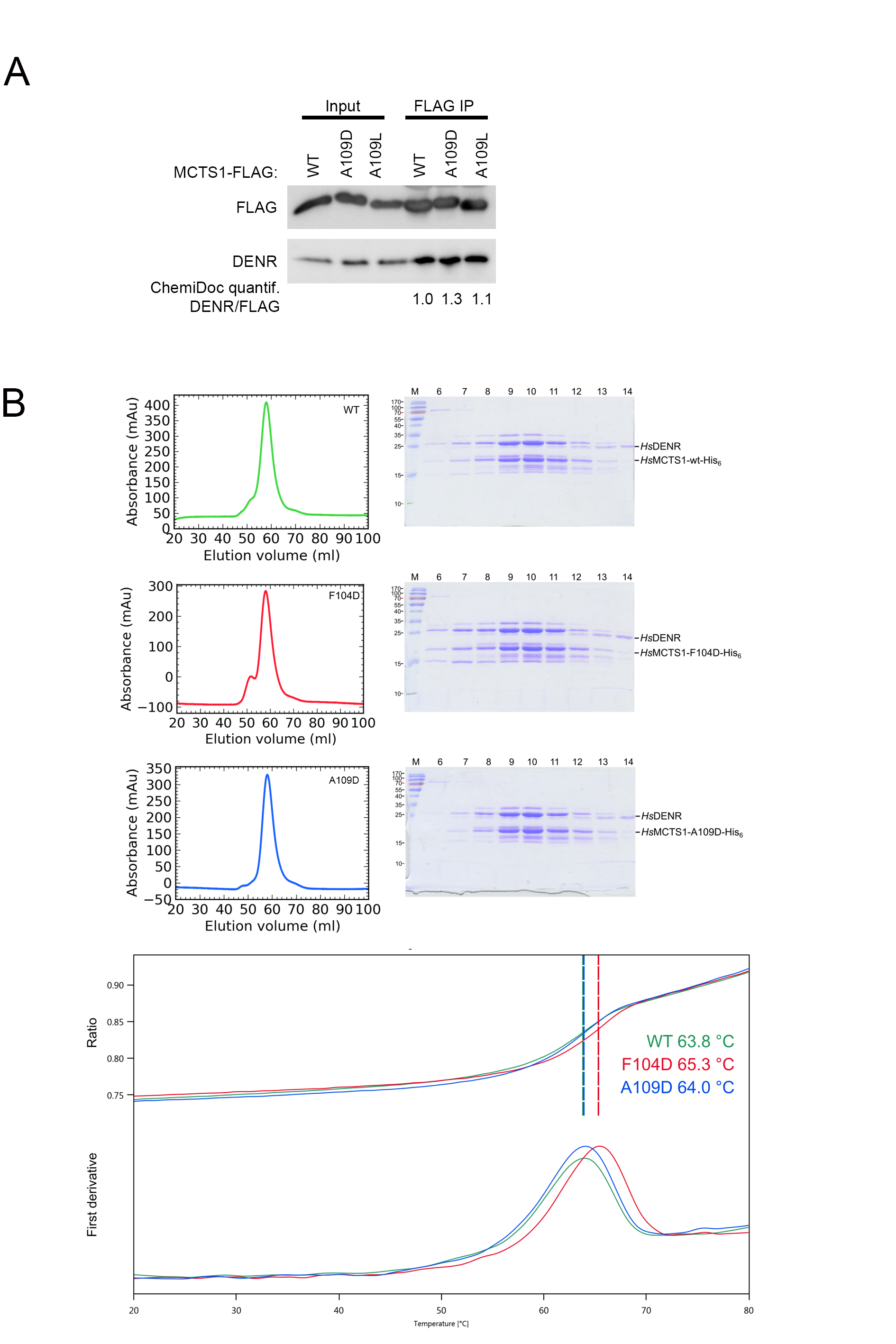

Supplement: S4 Fig — (A) MCTS1[A09D] and MCTS1[A109L] bind endogenous DENR. FLAG-tagged WT and mutant MCTS1 were expressed in HeLa cells and immunoprecipitated using the FLAG tag. Immunoprecipitates were probed for binding to endogenous DENR. Amount of coimmunoprecipitated DENR, normalized to the amount of FLAG–MCTS1, was quantified using the ChemiDoc system and is shown below the immunoblots. (B) Melting curves of WT (green) and mutant DENR–MCTS1 complexes (F104D and A019D, red and blue, respectively). The ratio between fluorescence counts at 350 and 330 nm is plotted against temperature (upper panel). Inflection points in the 350/330 ratio, which correspond to the melting temperature, are determined by the first derivative plotted against temperature (lower panel). The WT complex unfolds at 63.8°C, the F104D mutant at 65.3°C, and the A109D at 64.0°C. DENR, density-regulated reinitiation factor; MCTS1, multiple copies in T-cell lymphoma-1; WT, wild-type. (TIF) [file pbio.2005160.s004.tif]

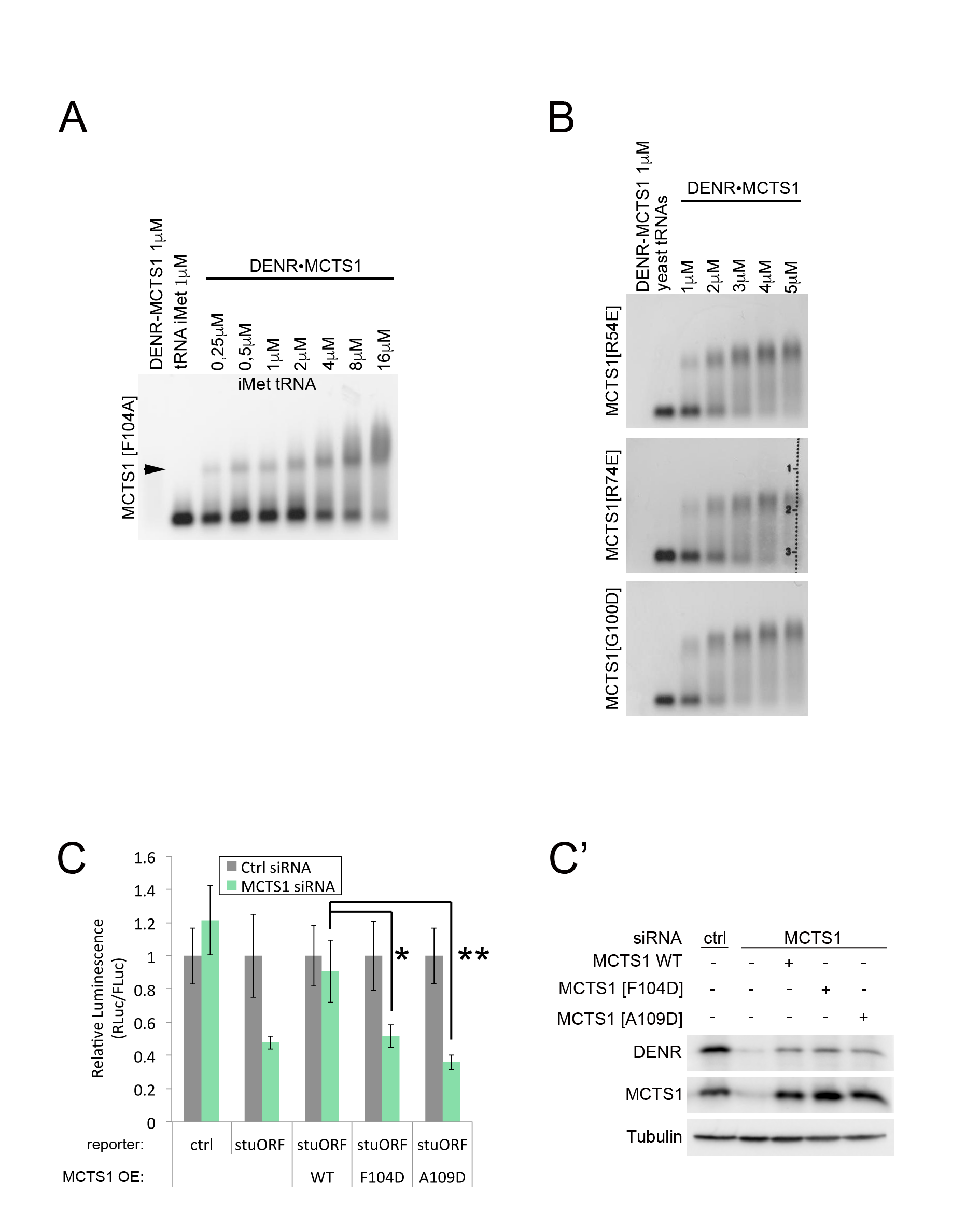

Supplement: S5 Fig — (A) The MCTS1 F104A mutation does not impair iMet-tRNA binding. tRNA binding assayed by gel shift assays as in S3 Fig. (B) Several mutations introducing negative charges on MCTS1 surface residues—such as R54E, R74E, and G100D—do not impair binding to yeast tRNA assayed by gel shift assays. (C-C') MCTS1 must bind tRNA to be functionally active. (C) Activity of MCTS1 tRNA-binding mutants, assayed by reconstituting MCTS1-knockdown HeLa cells with mutated MCTS1 overexpression constructs. Overexpression constructs also contain synonymous substitutions to avoid siRNA-mediated knockdown. Activity is assayed as the ability to promote translation reinitiation downstream of a stuORF as previously reported [10]. (C') MCTS1 protein levels from the same set of cells as in (C). Underlying data available in S1 Data. DENR, density-regulated reinitiation and release factor; MCTS1, multiple copies in T-cell lymphoma-1; siRNA, small interfering RNA; stuORF, upstream open reading frame with a strong initiation context. (TIF) [file pbio.2005160.s005.tif]

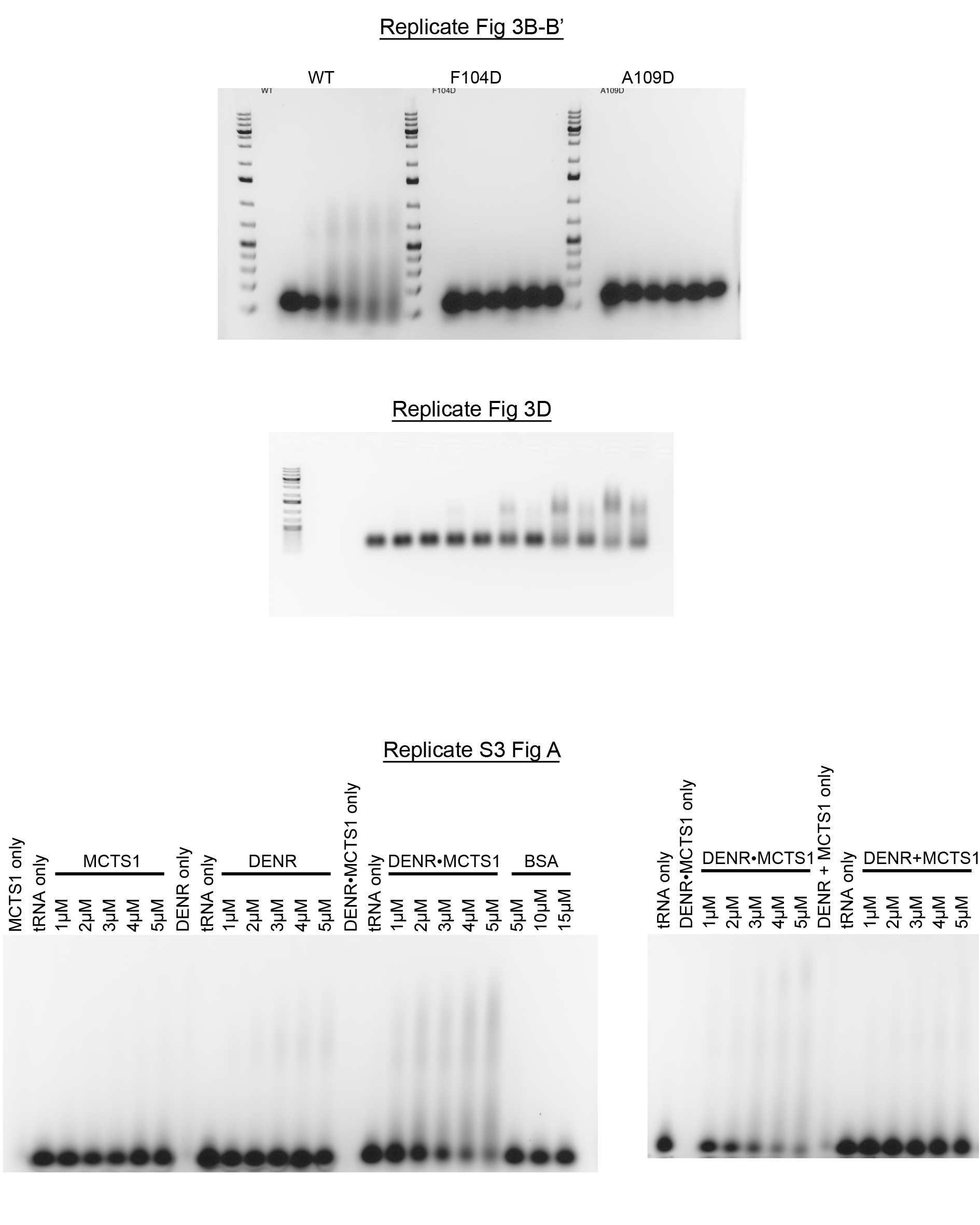

Supplement: S6 Fig — (TIF) [file pbio.2005160.s006.tif]

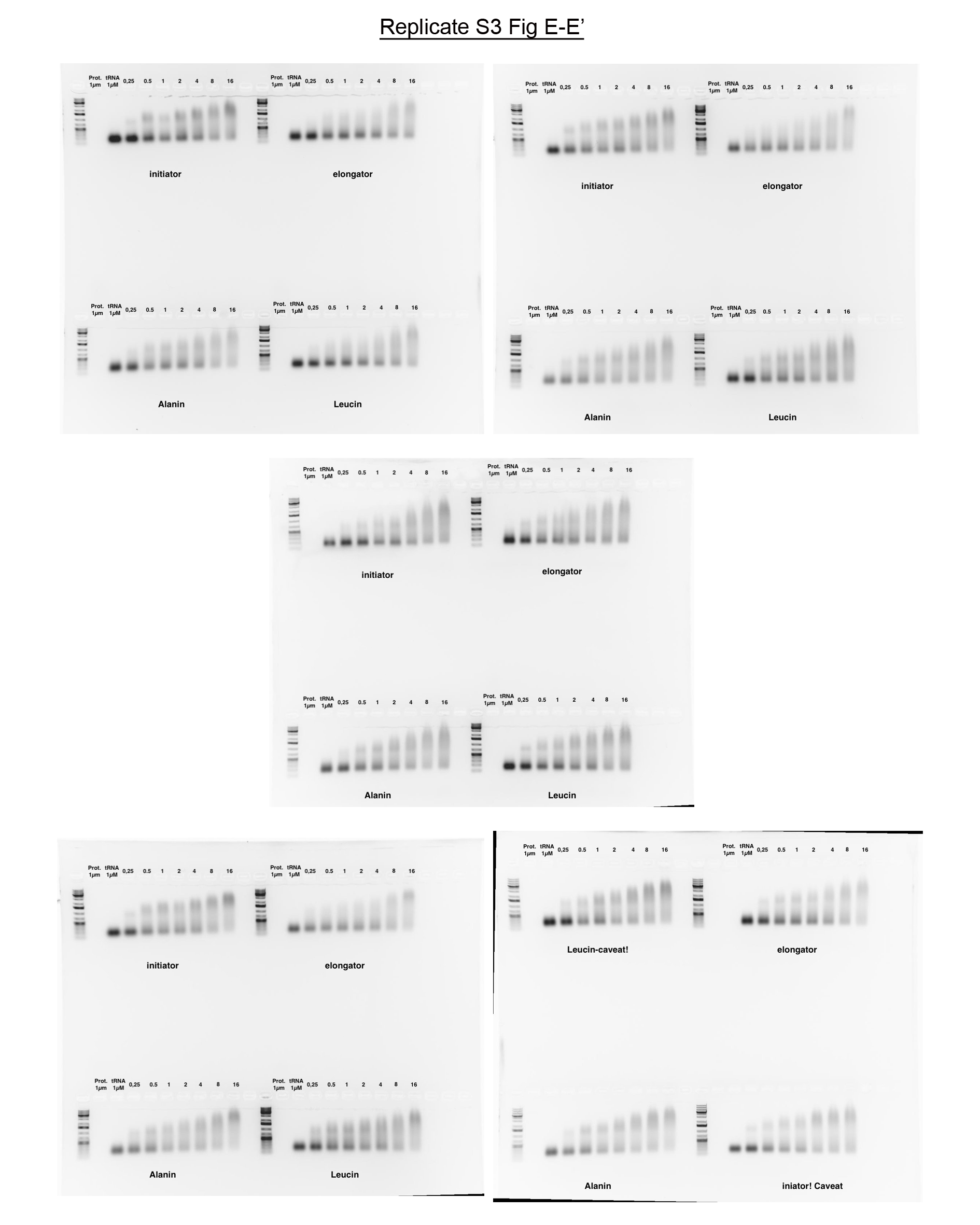

Supplement: S7 Fig — (TIF) [file pbio.2005160.s007.tif]
